# The Low Energy Availability in Females Questionnaire (LEAF-Q) as a Useful Tool to Identify Female Triathletes at Risk for Menstrual Disorders Related to Low Energy Availability

**DOI:** 10.3390/nu15030650

**Published:** 2023-01-27

**Authors:** Joanna Witkoś, Grzegorz Błażejewski, Marcin Gierach

**Affiliations:** 1 Faculty of Medicine and Health Science, Andrzej Frycz Modrzewski Krakow University, G. Herlinga-Grudzińskiego Street 1, 30-705 Krakow, Poland; 2 Department of Endocrinology and Diabetology, Collegium Medicum, Nicolaus Copernicus University, Skłodowskiej-Curie Street 9, 85-094 Bydgoszcz, Poland

**Keywords:** low energy availability, triathlon athletes, menstrual disorders, LEAF questionnaire

## Abstract

Background: Nutrition in sport is a priority; it is the basis for maintaining optimal health and a prerequisite for the high performance necessary for competitions. The aim of this study was to assess low energy availability and its possible consequences among female triathletes by using the Low Energy Availability in Females Questionnaire (LEAF-Q). Methods: The study involved 30 female triathetes. The LEAF-Q was used in the study. An analysis of the body composition was carried out with the seca device mBCA 515 medical Body Composition Analyzer. Results: Of the 30 female triathletes studied, 23.3% had a monthly cycle disorder, defined as an amenorrhea state for more than 90 days. No differences were found in injury rates or training days lost to injury due to menstrual disturbances. Menstruation changes were significantly greater due to increases in exercise intensity, frequency, and duration in the group experiencing menstrual disturbances (85.7 [95% CIs: 42.1–99.6] vs. 8.7 [95% CIs: 1.1–28.0]). The menstrual disorder group had a greater incidence of their periods stopping for more than 3 months than the group without menstrual disturbances. Conclusions: The female triathletes did not show abnormalities in body weight or composition, and these were not related to the incidence of menstrual disturbances. However, 20% of the triathletes either had, at the time of the study, or had had in the past monthly cycle disorders that could indicate an immediate risk of low energy availability. The LEAF-Q identified 10% of the triathletes as at risk (score > 8) of low energy availability and the physiological and performance consequences related to relative energy deficiency in sports (RED-S).

## 1. Introduction

The triathlon is a unique, extreme, long-distance endurance sport characterised by its multidisciplinary nature, in which swimming, cycling, and running are performed sequentially within the same sporting event. This type of sport undoubtedly presents a significant challenge to both the body and mind of the athlete [1]. The most popular standard triathlon distances are the sprint, i.e., 1/8 Ironman, which comprises back-to-back swimming 0.45 km, cycling 22.5 km, and running 5.25 km. The Olympic distance triathlon has a 1.5 km swim, 40 km cycle, and 10 km run; the 1/4 Ironman has a 0.9 km swim, 45 km cycle, and 10.55 km run; and the half-Ironman has a 1.9 km swim, 90 km cycle, and 21.1 km run. Particularly tough athletes can take part in the Ironman competition, which was introduced in 1978 on Waikiki Beach on the Hawaiian island of Oahu with just 12 participants [2]. Here, the distances consist of 3.8 km of open water swimming, 180 km of cycling, and 42.2 km of running to make up a total of approximately 8 to 17 h of continuous physical activity [3]. Since the first triathlon competitions, this endurance event has spread worldwide, attracting thousands of endurance-trained athletes. All competitions and their distances are regulated by the World Triathlon, the highest organisation in the world that oversees and regulates official triathlon competitions [4].

The Triathlon consists of the three above-mentioned sports disciplines, and in each of them, a low body fat percentage is an important factor for performance [5,6]. However, body fat is more of a predictor of ironman performance in men than in women [5,6]. Both male and female triathletes have very high training loads [6], which can make it difficult to match the energy intake with expenditure. Low energy availability (LEA) occurs when the energy intake from food is too low (insufficient) to cover both the energy needs of the sports training and the body’s basic physiological functions, including growth, immune, and reproduction functions, as well as thermoregulation [7,8,9]. An individual (athlete) exhibiting LEA therefore lacks the energy to sustain all normal bodily functions, and such a condition can occur with or without an eating disorder (ED) or disordered eating (DE) behaviour. LEA occurs for various reasons, but it is always based on not consuming enough food in relation to the energy requirements of the athlete’s body in a given period. Such a situation may be triggered consciously (food intake restriction) or unconsciously, as a result of a lack of knowledge of the proper nutrition that is required when undertaking physical activity. Psychological eating disorders such as anorexia nervosa or bulimia nervosa, which may or may not be related to sports, also contribute to the occurrence of LEA [7,8,9]. Low energy availability contributes to disruption in the body at both the physiological and psychological level, with consequent adverse effects on the athlete’s overall health and performance. Many studies [10,11,12,13] point to the fact that LEA is an underlying cause of both Female Athlete Triad (FAT) involving the interplay between eating disorders, disruption to or absence of a regular monthly cycle and low bone mineral density (BMD), and the syndrome of compromised body health resulting from an energy deficit, which is termed Relative Energy Deficiency in Sports (RED-S) by the International Olympic Committee (IOC) [13]. FAT and RED-S-related menstrual disorders, resulting from low energy availability, are called Functional Hypothalamic Amenorrhea (FHA), and the basis for these disorders is disruption to the hypothalamic–pituitary–gonadotropin axis (HPG) [14].

The organism of any human being and, in particular, the athlete undoubtedly requires an optimised diet. LEA initially leads to a negative energy balance manifesting itself in weight loss, but a prolonged lack of adequate food intake results in metabolic and physiological adaptations aimed primarily at survival, meaning that the athlete’s body acquires a new state of energy balance. The athlete’s body weight may be stable, but the body now has many impaired functions as a result of LEA [15,16,17]. The IOC consensus published in 2018 [13] described LEA-associated disorders that encompass a far more extended concept of health impairment than just FAT, which is only a small part of the RED-S concept in the overall picture reported by the IOC. The health consequences of RED-S include endocrine, immunological, haematological, and metabolic disorders; growth and developmental disorders; impairments to the cardiovascular function with unfavourable lipid profile and to the gastrointestinal functions; and finally, abnormalities in the psychological functions. The potential consequences of RED-S, though, are a decrease in endurance performance, muscle strength, glycogen stores, immune response, concentration, coordination, and training response [13]. In addition, the following have been reported: an increase in injury risk, stress fractures, osteoporosis, impaired judgement, depression, and irritability, together with a decrease in serum lipids and a decrease in glucose, blood pressure, and the resting metabolic rate [13].

In many scientific publications [18,19,20,21,22,23,24,25], LEA-related endocrine disorders have been described in both sportswomen and those women who do not practise sports. However, LEA is always a trigger for energy conservation via endocrine alterations. Among these are gonadotropin-releasing hormone (GnHR), ghrelin, adipokines, peptide YY (PYY), insulin, oxytocin, cortisol, growth hormone (GH), insulin-like growth factor 1 (IGF-1), triiodothyronine (T3), thyroxine (T4), and thyroid-stimulating hormone (TSH) [18,19,20,21,22,23,24,25]. A link has been confirmed between LEA and the altered pulsatile GnRH secretion, which, in turn, leads to a disruption in the secretion of the luteinizing hormone (LH) and follicle-stimulating hormone (FSH) by the pituitary gland. This reduces the secretion of oestradiol and progesterone by the ovaries, resulting in anovulation. Interestingly, different thresholds of energy deficiency affect the frequency of menstrual disturbances but not the severity [22,24]. Research showed [22,24] that there is no linear relationship between progressive energy deficiency and disturbances in the menstrual cycle. It has been found that pulsatile LH secretion is disrupted as soon as the energy level in a woman’s body drops below a certain level. This level has been reported as <30 kcal/kg FFM. Adequate energy availability (EA) is defined as the difference between energy intake and energy expenditure required for exercise in relation to fat-free mass (FFM) [15,16]. The current thresholds of energy availability for physically active women include, optimally, i.e., when the level of energy availability is at least 45 kcal/kg FFM/day, and this is recommended for maintaining all physiological functions. Another threshold is subclinical LEA, where EA is between 30 and 45 kcal/kg FFM/day. This amount of energy is permissible in a controlled manner and for a short period of time, e.g., when the aim is weight loss as part of a well-constructed diet and exercise [26]. Finally, clinical LEA occurs when energy falls below 30 kcal/kg FFM/day, and this level is too low for optimal body function and maintenance of satisfactory sports performance. Research [27] with eumenorrheic women showed that barely five days with an energy availability lower than 30 kcal/kg FFM/day induced severe endocrine and metabolic changes. The nutritional needs of an athlete necessitate both meeting the basic dietary requirements of the body and achieving sport-specific dietary goals. This means that the amount of energy provided by food must be high enough to provide the resources for the proper maintenance of basic metabolic functions and to secure the training loads occurring, depending on the requirements of the particular sport’s season [27,28,29]. To sum up, the reduced content of adipose tissue in the body of, for example, female athletes may negatively affect the correct hormonal activity of this tissue, which is essential for women and their reproductive functions [22]. However, body fat measurements alone will not predict athletes at risk for LEA and RED-S, and therefore, another method needs to be found as a screening tool.

Questionnaires can conveniently identify the symptoms of LEA and/or RED-S risk factors [7]. There are various screening tools to detect athletes at risk of LEA, e.g., Brief Eating Disorder in Athletes Questionnaire (BEDA-Q), Eating Disorder Examination Questionnaire (EDE-Q), Eating Disorder Inventory (EDI)–Drive for Thinness (DT) score, Eating Disorder Screening for Primary Care (ESP), Female Athlete Triad Risk Scale, Female Athlete Triad Screening Questionnaire, Female Athlete Screening Tool (FAST), Meal attitudes and body weight questions, RED-S risk measurement for cyclists, RED-S Specific Screening Tool (RST) (female and male versions), RED-S Specific Screening Tool (RST) (female and male versions), Three-Factor Eating Questionnaire (TFEQ)–Dietary cognitive restraint, Triad consensus panel screening questions by the Female Athlete Coalition, American Physiological Screening Test for eating disorders among Female College Athletes (PST), and Eating Attitudes Test (EAT)-26 [7]. Melin et al. [30], in 2014, published the Low Energy Availability in Females Questionnaire (LEAF-Q). The LEAF questionnaire is used to identify those female athletes who may be at risk of FAT syndrome and includes questions on injuries, gastrointestinal, and reproductive functions. It was developed as a screening tool to enable the self-reporting of persistent energy deficiency in female athletes. The questionnaire allows an early diagnosis of FAT and RED-S even where changes to the bone mineral density and body composition or other variables such as physiological symptoms linked to long-term low energy availability have not yet occurred. LEAF-Q has a 90% specificity and 78% sensitivity in successfully identifying FAT/RED-S. This tool identified eight out of ten female athletes with low EA and/or oligomenorrhoea/FHA and/or low BMD, as well as nine out of ten athletes with higher EA, eumenorrhea, and normal BMD. Often, gastrointestinal problems are an issue for female endurance athletes, as well as anyone with disordered eating/eating disorders. The variable for gastrointestinal problems was corroborated by the current lower availability of energy. When there is persistent energy deficiency, mucosal atrophy occurs; typically, this leads to diminished intestinal function and certain morphological changes linking current lower EA to gastrointestinal problems [30]. Restricted eating behaviour and menstrual dysfunction can lead to an increased risk for muscular skeletal injuries in female athletes. Research [30] confirmed that when female athletes have restricted eating behaviour, menstrual dysfunction, and low BMD, there is an increased risk of muscular skeletal injuries. LEAF-Q complements other eating disorder screening tools in enabling early detection and intervention for those female athletes at risk of FAT [30]. LEAF-Q is easy and inexpensive to implement across a variety of sports. It was used with different groups of athletes such as elite runners [31], elite and recreational female aesthetic sports athletes [32], elite rock climbers [33], female recreational exercisers recruited via gyms and fitness centres [34], football players [35], paralympic athletes [36], American football players [37], and ultra-marathon athletes [38].

To the best of the authors’ knowledge, LEAF-Q has not been used in triathletes, and that is why this investigation was carried out. The article adds to the literature on examining ways to assess athletes suffering from RED-S. The aim of this study was to assess the incidence of injuries, gastrointestinal problems, and disruption to the monthly cycle among female triathletes using LEAF-Q. In addition, quantitative measurements were made of the body composition of the athletes. The detailed research hypotheses were as follows. Female triathletes have high training loads that can lead to LEA. Female triathletes are at risk of FAT. Body composition and body weight do not necessarily identify female triathletes at risk of RED-S. Women experience menstrual changes when their exercise intensity, frequency, or duration is increased. LEAF-Q is a useful screening tool for the early detection of FAT symptoms.

## 2. Materials and Methods

### 2.1. Participants

The study involved 30 female triathetes from the Kujawsko-Pomorskie and Wielkopolskie Voivodeships in Poland. The athletes were affiliated to sports clubs, and the training sessions were conducted by professional trainers. The women had been training for an average of 6.0 ± 2.8 years. The number of hours a week spent on training by the participants was 6.7 ± 1.6 h, with a single workout lasting 75.0 ± 21.2 min. The training sessions of the female participants occurred every week and were mixed in nature, taking place between four and six days a week. Swimming training took place twice a week with an average duration of 52.5 ± 10.6 min, as did cycle training with a duration of 90.0 ± 42.4 min. Running sessions took place four times a week with a duration of 75.0 ± 21.2 min. The women mainly competed in 1/2, 1/4, or 1/8 Ironman distances. A sample training plan included: on Monday, a leisurely run referred to as BC1, covering approximately 10–12 km; Tuesday morning (7 am–8 am), pool training, and afternoon bike training, including a 15 min warm-up, followed by 20 × 1 min intervals at the appropriate heart rate range: as a rule, one minute hard/one minute light with 20 repetitions, i.e., a total of 40 min with a 15 min rest period. Wednesday was a day off. Thursday included an eight-to-ten-kilometre intense run at a heart rate of 75–85% of the maximum heart rate, preceded by a ten-minute warm-up and the same amount of rest. Friday morning, the athletes undertook pool training (7 am–8 am), followed in the afternoon by a light BC1 10–12 km run. Saturday was a day off. Sunday, there was 90 min bike training, followed by a one-hour run, including a 15 min warm-up, 10 × 150 m ascents and 50 m returns, and a 100 m light run, with the last 15 min being a cooldown.

In all the female athletes, the first menstruation occurred naturally at an average age of 12.3 ± 1.9 years. However, it should be noted that about half of the women (56.7%) had menarche between 12 and 14 years of age; in 33.3%, it occurred under 11 years of age; and in 10.0%, over 15 years of age. Inclusion criteria for the study were regular triathlon training and participation in Ironman competitions. Women using hormonal contraception and those who did not complete the questionnaire correctly were excluded from the study.

### 2.2. Experimental Design

#### 2.2.1. Body Composition Analysis

Analysis of the body composition of the female athletes studied was carried out with the seca device mBCA 515 medical Body Composition Analyzer. This machine was chosen for the study because it had undergone an extensive scientific validation process [28,29]. The device measures the composition of the body by bioelectrical impedance analysis using a pair of electrodes for each hand and foot. The tests were performed after 8 a.m., and the participants had been fasting. They were informed in advance and asked not to drink any fluids before the tests on the day, although they were asked to hydrate the day prior to testing. The subjects were asked to refrain from training prior to the body composition testing. Body weight and height were determined without shoes and in minimal clothing. This condition was essential to obtain accurate and reliable results from the body composition analyser.

#### 2.2.2. Questionnaire

LEAF-Q was used in the study (Supplement S1). The LEAF-Q was created with the aim of identifying female athletes ‘at risk’ of the physiological symptoms associated with LEA, and, thus, the risks of LEA [30]. The LEAF-Q includes 25 questions arranged in three separate, sequential sections: injuries, gastrointestinal, and reproductive functions. A list of questions was scored using the LEAF-Q Scoring Key (Supplement S2). The LEAF-Q questionnaire authors’ suggested cut-off values for injury, gastrointestinal disorders, and menstrual disorders are ≥2, ≥2 and ≥4, respectively. A total score of ≥8 out of 25 questions indicates that the competitor is at risk of LEA and, as a consequence, may be at risk of developing the FAT and RED-S groups of symptoms [30].

### 2.3. Statistical Analyses

Statistical analyses were performed using the SPSS 27 software program (Version 27.0, IBM Corp., Armonk, NY, USA). The tests used included the following: the Mann–Whitney *U* (this nonparametric test was used as most variables were not normally distributed) and the chi-square test. The alpha level for all analyses was 0.05. Data were reported as means ± standard deviations. As for effect sizes, for continuous variables, Cohen’s d was calculated as the effect size, and for categorical ones, the following was included: Cramer V. Following the guidelines for the interpretation of effect sizes, the following were assumed [39] for Cohen’s d: 0.2, 0.5, and 08; for Cramer V, with 1 df: 0.1, 0.3, and 0.5; and for Cramer V with 2 dfs: 0.07, 0.21, and 0.35 for small, medium, and large effect sizes, respectively.

## 3. Results

The mean age of the triathetes was 33.5 years ± standard deviation 9.2 years (*Me* = 31.5). The average height of the women was 169 ± 5.9 cm (*Me* = 170), body weight was 61.7 ± 9.5 kg (*Me* = 61.3), and body mass index (BMI) was 21.5 ± 2.5 kg/m^2^ (*Me* = 21.4).

Secondary amenorrhea is defined as a state of amenorrhea lasting more than 90 days [10,11]. Therefore, for further analysis, individuals were divided into two groups. The division criterion was a LEAF questionnaire question: “Have your periods ever stopped for 3 consecutive months or longer (besides pregnancy)?”. This identified the athletes who had menstrual cycle disorders over 3 months (“problem”) and those who did not have such problems (“no problem”).

The Body Composition Analysis for the entire group was as follows: the average fat mass (FM) expressed in kilograms 16.4 ± 4.9 kg (*Me* = 15.4), the percentage of FM 26.0 ± 5.2% (*Me* = 24.3), the abdominal visceral adipose tissue (VAT) at 0.4 ± 0.2 (*Me* = 0.4), fat-free mass (FFM) in kilograms 45.3 ± 6.6 kg (*Me* = 43.5), lean mass as a percentage 73.7 ± 5.2% (*Me* = 75.3), muscle mass (MM) in kilograms 20.8 ± 3.9 kg (*Me* = 19.4), total body water (TBW) 33.7 ± 5.6% (*Me* = 31.6), and extracellular water (ECW) 14.7 ± 1.9% (*Me* = 13.9). The ratio of ECW to TBW was 42.5 ± 1.6%. A detailed list of the above parameters, divided into women who declared monthly cycle abnormalities and those who did not, is presented in Table 1.

An analysis of the results obtained, in terms of the monthly cycle disorders present in the triathletes surveyed found that 23.3% of the women had a monthly cycle disorder. An analysis of the answers provided by the triathletes to the LEAF questionnaire found that a result of ≥8 out of 25 questions was achieved by three female athletes (10%). A further three athletes (10%) scored seven points. A total score of 5 was obtained by a further five athletes, which accounted for 16.7%.

When considering in detail the questions on menstrual function obtained from the questionnaire, it was found that an uninterrupted monthly cycle was declared by 76.7% of the women, while seven female athletes (23.3%) stated that their monthly cycle was not regular, i.e., did not occur every 28th to 34th day (Table 2). Six female athletes, which accounted for 96.7% of those who reported abnormalities in the menstrual cycle, had no menstrual bleeding for a period lasting 2 to 3 months, and one woman (3.3%) had no menstrual bleeding for 4 to 5 months. Some (13.3%) patients reported an occurrence of no menstrual bleeding in the past, while 10.0% of the triathletes had no menstrual cycle at the time of the test. When the subjects were asked if anything changed in their monthly cycle with an increase in intensity, frequency, or duration of training, 26.7% said that they experienced less bleeding than normal when there was an increase in physical exertion.

The incidence of sports injuries in the last year was declared by eleven (36.7%) of the women. Among these triathletes, there were three, which constituted 27.3%, who had reported disorders of the monthly cycle in the research. Their results in the questionnaire were eight points for two of them and seven points for one, i.e., significant enough to talk about the risk of LEA and, thus, FAT and RED-S development. The number of days absent from training among the injured women, i.e., eleven athletes, was as follows: one to seven days of absence from training occurred in 50.0% of the women with a normal monthly cycle and 66.7% of women with disorders of the monthly cycle; eight to fourteen days of absence from training occurred in 37.5% of triathletes with a normal cycle and 33.3% with an abnormal cycle; and an over 22 days of absence from training occurred in only one athlete, and she had a normal cycle (12.5%). No significant disorders related to gastrointestinal function were found in any of the athletes. Responses to the LEAF-Q questionnaire by women with and without a monthly cycle disorder are shown in Table 2.

## 4. Discussion

In this study an assessment of relative energy deficiency using the LEAF questionnaire showed low energy availability in seven of the women (23.3%), as evidenced by the presence of monthly cycle disorders. It should also be noted that three women did not have a regular monthly cycle at the time of the study. It can therefore be said with a high degree of certainty that these women are suffering from LEA [9,10,11,12,13]. Answers provided by the triathletes to the LEAF questionnaire found that a result of ≥8 out of 25 questions was achieved by three female athletes. This indicated that they were at immediate risk of LEA and, as a consequence, symptoms of FAT and RED-S syndrome could soon arise. A further three athletes scored seven points. These women also have a relatively high risk of either developing, or, at least, seeing the symptoms of FAT and RED-S in the near future. A further five athletes scored five points, and this is high enough to pre-suppose that there is a risk that this score could well increase over the following months.

Sports injuries occurring in the last year among the female athletes were recorded in approximately 35% of the women, i.e., 8 out of 23 athletes, with a normal monthly cycle and approximately 43% of the women (3 out of 7 athletes) with a disrupted monthly cycle. The possible lack of oestrogen, due the absence of a regular monthly cycle, can lead to a decrease in BMD and an increased risk of injuries, especially stress fractures [9,10,11,12,13]. In a study by Jusus et al. [31] conducted among elite runners, it was found that over the past year, stress fractures were the third-most reported injury among these athletes and that these affected 2.25% of women. It was also noted that athletes of both sexes who reported stress fractures, usually associated with reduced BMD, were identified as being at risk of LEA. Meng et al. [32] studied the risk of LEA among elite and recreational female aesthetic sports athletes. They found that female athletes, who were at risk of LEA, were more likely to have sports injuries compared to those female athletes who were not at risk of LEA, 62.5% vs. 16.7% respectively. There was also a difference in the incidence of sports injuries between elite aesthetic sports athletes and recreational aesthetic athletes of 30.8% vs. 23.7%, respectively. A study by Condo at al. [35] found that female football players, who were classified as being at risk of LEA, reported sports injuries occurring in the last year. For example, the percentage of training days lost due to injuries lasting 15 to 21 days was 11%, with a similar percentage for those injuries more than 22 days. However, a higher percentage of lost training days was reported by Łuszczki et al. [40], who also studied football players. The number of lost training days due to injury of 15 to 21 days was 14.7%, and above 22 days, it reached 20.6%. Folsher et al. [38], studying ultra-marathon athletes, reported an incidence of stress fractures in 3.4% of female participants. In contrast, the number of days lost to injury treatment was 7.8% for 15 to 21 days and 10.5% for recovery beyond 22 days.

Body composition assessment is widely used in clinical practice for nutritional evaluation and monitoring, such as for investigating obesity or malnutrition, as well as for evaluating athletic health and performance [29,41,42]. Accurately and reliably assessing the various components of body composition helps to monitor the appropriateness of the training and optimise the performance of the athlete. Excess FM is generally perceived by athletes as a major limiting factor in sporting performance, while increased skeletal muscle mass can promote the development of strength and power. Athletes have a greater FFM than those who do not undertake training [43]. Athletes are interested in achieving an optimal balance between FFM, FM, and total body mass appropriate for their particular sport. A higher FM can have an adverse effect on performance in weight bearing and aesthetic sports including running, gymnastics, and the triathlon among a few [30,41,42]. Moreover, there has been a growth in assessing other body composition variables, such as total body water (TBW), intracellular water (ICW), and extracellular water (ECW), in order to monitor the hydration status in athletes [43]. Santos et al. [44] provided reference values for body composition and anthropometric measurements in athletes involved in a total of 21 sports. Sex- and sport-specific reference percentiles were developed for body composition at both the molecular and whole body level, respectively, using dual-energy X-ray absorptiometry (DXA) and anthropometry. The authors observed sex differences for all variables, as well as differences by sport. As the research for this article only included female triathletes, the following comparison focuses exclusively on women. Santos et al. [44] indicated, that triathletes’ respective age by sport (range) was 16–27 years, mean 21.0 ± 3.5 years; however, the age for the women in this research was 33.5 ± 9.2 years. The consecutive reference values for the main anthropometry results were body mass, mean 57.9 kg (53.5–62.3) vs. competitors tested in this research 61.7 ± 9.5 kg, height 168.8 cm (163.8–173.0) vs. 169 ± 5.9 cm, and BMI (kg/m^2^) 20.4 (19.1–21.8) vs. 21.5 ± 2.5 kg/m^2^. The average fat mass (FM) expressed in kilograms was 11.4 kg (8.2–14.7 kg) vs. 16.4 ± 4.9 kg with percentages of fat mass 20.0% (15.6–24.4%) vs. 26 ± 5.2%. Fat-free mass (FFM) was 44.7 kg (42.8–46.6 kg) vs. 45.3 ± 6.6 kg for the tested women. When comparing the above data from our own research, it can be concluded that the figures are close to the reported reference values for the sport, i.e., the study population was a good representation of female triathletes [44]. The reference value for female triathletes with menstrual problems tested in this research were also similar to the group studied by Santos et al. The average age for female triathletes with menstrual problems was 36.9 ± 10.3 years, body mass 61.7 ± 7.4 kg, height 168.9 ± 4.6 cm, BMI (kg/m^2^) 21.5 ± 1.9 kg/m^2^, the average FM expressed in kilograms 16.1 ± 2.3 kg, with percentages of 24.7 ± 4.8%, and FFM was 47.0 ± 6.0 kg. It should be emphasised that there was no difference in the body weight and composition between the groups and thus these would not be a valuable measure for determining RED-S risk. Other reference values for the female triathletes provided by Santos et al. [44] were as follows: fat mass index (FMI) kg/m^2^ mean 4.2 (2.8–5.5) and fat-free mass index (FFMI) (kg/m^2^) 16.1 (15.2–17.0). Lean soft tissue (LST) was 42.7 kg (40.9–44.6 kg), appendicular lean soft tissue (ALST) 18.8 kg (17.7–20.0 kg), and appendicular lean soft tissue index (ALSTI) kg/m^2^ mean 6.78 (6.3–7.3) [44].

Mass participation in triathlon is a new trend that manifests itself as the human need to seek strong emotions in a difficult, extreme sport [45]. The complexity of the triathlon and its multidisciplinary nature means that it includes workouts tailored to three different sports, which also affects the type of diet triathletes should eat. The number of training sessions undertaken by a triathlete is significantly greater than that undertaken by those competitors training in only one sport discipline, so this should also translate into adequate energy consumption. The high number of calories expended should be matched by the need for a high calorie intake to match that expenditure. Many athletes do not realize how many calories are needed to maintain energy availability and thus end up experiencing LEA and its consequences. For example, a world-class female athlete completed 796 sessions over 50 weeks, or about 16 training sessions a week, preceding the 2012 London Olympics [46,47]. The proportion of women participating in the triathlon has increased over the last three decades and they now account for 25–40% of all competing athletes [48].

The complexity of the triathlon and its multidisciplinary nature means that training session durations and intensities and the energy intakes required are significantly greater than that undertaken by competitors training in only one sport discipline. Many triathletes do not realize how many calories are needed to maintain energy availability and thus end up experiencing LEA and its consequences. For example, a world-class female triathlete completed 796 training sessions over 50 weeks, or about 16 training sessions per week, preceding the 2012 London Olympics [46,47]. The proportion of women participating in the triathlon has in creased over the last three decades and they now account for 25–40% of all competing athletes [48]. Athletes, as a group of people whose anthropometric characteristics affect the results achieved in a specific sport, are particularly exposed to DE, as they pay particular attention to their body weight [49]. Pathogenic eating behaviour has been shown to occur far more frequently in athletes than in non-athletic groups. Women, in particular, are at risk of DE. For example, in a study by Bratland-Sanda et al. [50] it has been shown that up to 95% of cases of DE affecting groups of athletes occur in women, 90% of whom are 25 years old or younger. The prevalence of eating disorders was also found to be higher in female athletes compared to male athletes, ranging from 0 to 19% in men and 6 to 45% in women. Assessing the energy availability in triathletes cannot be accomplished by body composition alone, and thus, questionnaires were developed to help.

The anthropometric characteristics of the women studied were similar to the values presented by other authors [51,52,53,54]. Most of the studies that have analysed data from elite triathletes have focused mainly on the athletes’ height, body weight, and body fat levels expressed as percentage body fat or total body skinfolds. Elite triathletes tend to have about 7–12% more body fat than those men taking up this type of sport [51,52]. The average height of female athletes has remained around 167 cm over the years, but a decrease in body weight has been noted, from around 60 kg at the beginning of the century to around 55 kg now. In comparison, the women who participated in our study, had a body weight which was on average 7 kg higher than today’s standard, while their average height of 169 cm was comparable. The female triathletes studied were on average 33.5 years old, which also brings them closer to the current standard of athletes representing the sport. As research by Malcata et al. [53] and Werneck et al. [54] showed, the optimal age for undertaking triathlon and the age at which maximum performance is achieved for women, is 27 ± 4 years. Similarly, Knechtle et al. [54] reported an optimal age of 26.6 ± 4.4 years for women.

This was the first time triathletes have been studied using LEAF-Q, although this questionnaire has been used with other groups of athletes. In the study by Jesus et al. [31], a total of 66 female elite runners (79.5%) were identified as being at risk of LEA, with 41.3% reporting irregular menstruation, according to LEAF-Q scores. When asked if their menstruation had ever stopped for at least three consecutive months, 32.5% reported past such experiences and 25.0% reported experiencing it at that moment. Additionally, 50% experienced changes in their menstruation when their exercise load increased [31]. The research [32] showed that, among female athletes from six sports (trampolining, rhythmic gymnastics, aerobics, dance sport, cheerleading and dance), a total of 41.6% of participants (*n*  =  69) were at increased risk of LEA, and 57.2% of participants (*n*  =  95) were classified as at high risk of eating disorders. For female elite athletes vs. recreational athletes, there was a significantly higher prevalence of LEA risk (55.8% vs. 35.1%), and amenorrhea (53.8% vs. 13.3%) [32]. In the study by Monedero et al. [33], eleven female advanced, or elite, rock climbers completed the Eating Attitudes Test (EAT)-26 and the LEAF-Q. Four female subjects were at high risk of LEA according to the LEAF-Q scores. In the study by Slater et al. [34], 109 female recreational exercisers, with a mean age of 23.8 years were recruited via gyms and fitness centres throughout New Zealand. A total of 45.0% of participants were classified as “at risk” of LEA. In a study [35] aiming to assess nutritional intake, sports nutrition knowledge and the risk of LEA in female Australian rules football players, the risk of LEA was evident in 30% of players. In another study [36] examining the symptoms of LEA and risk of RED-S symptoms in paralympic athletes, 78% of females were “at risk” of LEA using the LEAF-Q. In the studies by Magee et al. [37], LEAF-Q classified 56.3% of American football players as at risk of LEA. In a further study [38] of 306 athletes, who participated in ultra-endurance events, 44.1% were found to be at risk of FAT, and only 7.5% knew about the triad. Compared to our own study presented above [31], the percentage of females practising running and at risk of LEA was significantly higher: 79.5% vs. 23.3% in the triathletes studied. The problem of a disrupted monthly cycle did not occur in 38.2% of the female runners, which indicates that the monthly cycle can be maintained despite LEA. However, these cycles may be non-ovulatory when energy deficiencies occur [31]. Comparing the results of LEA in aesthetic sports with our own study, the risk of LEA was 41.6% of these women vs. 23.3% of female triathletes. Aesthetic sports require an ideal body shape, so, the almost doubled percentage of women with LEA in these sports when compared with the results of our own study may be due to the specific nature of the sport and the restrictive requirements of the weight categories, which force female athletes to undertake restrictive diets. It is also unsurprising that elite athletes showed a significantly higher percentage of menstrual cycle disorders than women undertaking aesthetic sports at a recreational level. This percentage was also lower than in our own study 13.3% vs. 23.3% among the triathletes [32]. The percentage of women at risk of LEA in our study was similar to that of Australian rules football players [35] at 23.3% vs. 30%, but interestingly, among female American football players [37] it was much higher at 56.3%. The differences may be due to a number of variables, such as the age of the women, training load and nutrition. Triathlon is a sport that puts a lot of strain on the athlete’s body, however, it is possible that the lower percentage of women at risk of LEA in our study compared to other sports was because the women studied were adopting the right dietary behaviours. These protected most of them from LEA, while still maintaining the adequate levels of body fat required to maintain reproductive function.

Nutritional and metabolic disorders have many negative consequences for women’s health, including the suppression of the reproductive functions, which are particularly sensitive to the nutritional status of the body. It seems that eating disorders which occur in athletes are mainly related to their inadequate education in this area or poor understanding of nutritional and energy requirements that apply when undertaking physical activity. Weight class restrictions and aesthetic requirements for particular sports can also influence an athlete’s decision about certain nutritional practices [55,56,57,58]. Restrictive diets, a drastic reduction in body weight and pathological eating behaviours can lead to the occurrence of LEA, and consequently to the further negative health consequences of FAT and RED-S. Early detection and treatment of athletes, who have eating disorders or who are unaware of the energy intake needed to match energy expenditure, and are, therefore, potential candidates for LEA, can help prevent the serious health consequences discussed in this paper [59]. However, early identification of those athletes suffering from energy deficiencies can be difficult due to the initial lack of obvious and clear symptoms of LEA, or because women treat the lack of monthly bleeding as opportune, giving them the chance to continue training without interruption. The use of simple and clearly interpretable questionnaires, such as the LEAF-Q, can be the basis for initiating action to reduce and prevent the serious consequences of LEA. If LEA is diagnosed too late, this can lead to serious health repercussions, with treatment lasting for a length of time during which the athlete loses the opportunity to undertake training, with obvious repercussions for their sporting career.

Limitations. The main limitation of the study was the relatively small group of women studied, but with this type of extreme sport, it was a significant challenge for the researchers to be able study even such a small group. The observed power was above 0.8 only with the following questions: Do you get cramps or stomach-aches that cannot be related to your menstruation? Have your periods ever stopped for 3 consecutive months or longer (besides pregnancy)? Does your menstruation change when you increase your exercise intensity, frequency, or duration? If yes, how? In the case of the remaining questions, the observed power was rather low, which is a limitation of this study.

Another limitation of the study was the fact that the research was carried out at only one time point, so it is not known whether the triathletes whose results in the LEAF-Q questionnaire indicated they were close to the risk level for the development of LEA, i.e., seven or even five points out of 25, would go on to register the higher LEA risk level of eight points after further months of training. The studied athletes were in their base training phase of the season. Conducting the LEAF-Q during different training phases (base, competition) would be very informative. It would also have been beneficial to the study to monitor the levels of selected hormones and to confirm the LEA, as well as determining the individual energy balance of the female triathletes. Adding blood markers such as cortisol, thyroid hormone, and leptin and bone turnover markers, as well as BMD from dual-energy X-ray absorptiometry (DXA) and the resting metabolic rate RMR, and then relating these to the LEAF-Q questions would also have been interesting.

## 5. Conclusions

The use of the LEAF questionnaire in the study allowed the early detection of FAT symptoms in several of the female triathletes studied. The female triathletes did not show abnormalities in body weight or composition, and these symptoms were not related to the incidence of menstrual disturbances. However, 20% of the triathletes either had, at the time of the study or had had in the past, monthly cycle disorders that could indicate an immediate risk of LEA. The LEAF-Q identified 10% of the triathletes as at risk (score > 8) for LEA and the physiological and performance consequences related to RED-S. The number of reported injuries in female triathletes in the last year was higher in those women with menstrual disorders compared to those women without, which may indicate that a lack of oestrogen can lead to an increased risk of injuries.

Nutrition in sports is a priority. It is the basis for maintaining optimal health and a prerequisite for the high performance necessary for competitions. However, when it comes to a successful performance and health, not only is good nutrition significant but also energy availability and the importance of energy intake matching the energy expenditure. Therefore, the results of the presented study examining LEA with LEAF-Q are important and should be taken into consideration by both athletes and the training staff.

## Figures and Tables

**Table 1:** Anthropometric and body composition data categorized by menstrual disturbance.

	Mean		Median		SD				
	No Problem (*n* = 23)	Problem (*n* = 7)	No Problem (*n* = 23)	Problem (*n* = 7)	No Problem (*n* = 23)	Problem (*n* = 7)	*U*	*p*	*d*
Age [years]	32.5	36.9	31.0	32.0	8.8	10.3	59.5	0.302	−0.47
Height [cm]	169.0	168.9	170.0	169.0	6.3	4.6	72.0	0.676	0.03
Weight [kg]	61.7	61.7	61.3	61.4	10.1	7.4	77.0	0.864	<0.01
BMI [kg/m^2^]	21.5	21.5	21.2	21.5	2.7	1.9	73.0	0.713	−0.01
FM [kg]	16.5	16.1	15.3	16.1	5.5	2.4	66.0	0.477	0.09
FM [%]	26.4	24.7	24.7	23.7	5.3	4.8	66.0	0.477	0.32
VAT [I]	0.4	0.3	0.4	0.3	0.2	0.2	57.0	0.240	0.57
FFM [kg]	44.8	47.0	43.3	43.6	6.8	6.0	61.0	0.339	−0.33
FFM [%]	73.7	73.6	75.3	75.3	5.3	5.1	78.0	0.902	0.03
MM [kg]	20.5	21.7	19.3	19.6	4.0	3.7	66.5	0.492	−0.30
TBW [%]	33.4	34.7	31.7	31.1	5.7	5.3	68.5	0.556	−0.22
ECW [%]	14.7	14.6	13.9	13.8	1.8	2.2	76.5	0.844	0.02
ECW/TBW [%]	42.6	42.1	42.4	41.7	1.7	1.5	74.0	0.750	0.30
No. of weekly training sessions	6.5	7.3	7.0	7.0	1.8	0.8	61.0	0.326	−0.48

SD—standard deviation, BMI—body mass index, FM—fat mass, VAT—visceral adipose tissue, FFM—fat-free mass, MM—muscle mass, TBW—total body water, and ECW—extracellular water.

**Table 2:** LEAF-Q results. OP: observed power. Numbers in parentheses: *N* needed for power = 0.8.

Low Energy Availability in Females Questionnaire	Menstruation			
Absences from your training, or participation in competitions during the last year due to injuries				
*chi^2^*(1) = 0.15; *p* = 0.698, C_v_ = 0.07 OP = 0.07 (1602)	No problem		Problem	
	N	%	N	%
No, not at all	15	65.2	4	57.1
Yes, once or twice	8	34.8	3	42.9
	23	100.0	7	100.0
Numbers of days’ absence from training or participation in competition due to injuries				
*chi^2^*(2) = 0.50; *p* = 0.780, C_v_ = 0.21 OP = 0.09 (219)				
1–7 days	4	50.0	2	66.7
8–14 days	3	37.5	1	33.3
15–21 days	0	0	0	0
22 days or more	1	12.5	0	0
	8	100.0	3	100.0
Do you feel gaseous or bloated in the abdomen, also when you do not have your period?				
*chi^2^*(1) = 0.31; *p* = 0.575, CV = 0.10 OP = 0.09 (785)				
Yes, once or twice a week or more seldom	1	4.4	0	0
Rarely or never	22	95.6	7	100.0
	23	100.0	7	100.0
Do you get cramps or stomach-ache that cannot be related to your menstruation?				
*chi^2^*(1) = 0.31; *p* = 0.575, C_V_ = 0.10 OP = 0.09 (785)				
Yes, once or twice a week or more seldom	1	4.4	0	0
Rarely or never	22	95.6	7	100.0
	23	100.0	7	100.0
When did you have your last period?				
*chi^2^*(1) = 3.40; *p* = 0.065, C_V_ = 0.34 OP = 0.34 (68)				
2–3 months ago	23	100.0	6	85.7
4–5 months ago	0	0	1	14.3
6 months ago or more	0	0	0	0
	23	100.0	7	100.0
Have your periods ever stopped for 3 consecutive months or longer (besides pregnancy)?				
*chi^2^*(2) = 30.00; *p* < 0.001, C_V_ = 0.99 OP > 0.99 (10)				
No, never	23	100.0	0	0
Yes, it has happened before	0	0	4	57.1
Yes, that’s the situation now	0	0	3	42.9
	23	100.0	7	100.0
Does your menstruation change when you increase your exercise intensity, frequency or duration?				
*chi^2^*(1) = 16.28; *p* < 0.001, C_V_ = 0.74 OP = 0.98 (15)				
Yes	2	8.7	6	85.7
No	21	91.3	1	14.3
	23	100.0	7	100.0
If yes, how?				
*chi^2^*(1) = 16.28; *p* < 0.001 OP = 0.98 (15)				
I bleed less	2	8.7	6	85.7
No changes	21	91.3	1	14.3
	23	100.0	7	100.0

## Data Availability

The datasets used and/or analysed during the current study are available from the corresponding author upon reasonable request.

## Supplementary Materials

The following are available online at https://www.mdpi.com/article/10.3390/nu15030650/s1. Supplement S1: The Low Energy Availability in Females Questionnaire (LEAF-Q); Supplement S2: The LEAF-Q scorring key.
