# Supplementary material for: The Low Energy Availability in Females Questionnaire (LEAF-Q) as a Useful Tool to Identify Female Triathletes at Risk for Menstrual Disorders Related to Low Energy Availability
Source: Nutrients. 2023 Jan 27;15(3):650. doi: 10.3390/nu15030650 (PMC9920150; doi:10.3390/nu15030650)
Supplement: Supplementary file 1 [file nutrients-15-00650-s001.zip › nutrients-2168140 - The LEAF-Q Scoring key.pdf]

(Supplemental Digital Content 2)

The LEAF-Q Scoring key

A total score ≥8 is to be considered at risk for the Triad

Department of Nutrition, Exercise and Sports
Life Science
University of Copenhagen
Denmark
Contact: Anna Melin, aot@life.ku.dk

1. A: **0** No, not at all, **1** Yes, once or twice, **2** Yes, three or four times, **3** Yes, five times or more

1. A1: **1** 1-7 days, **2** 8-14 days, **3** 15-21 days, **4** 22 days or more

2. A: **3** Yes, several times a day, **2** Yes, several times a week, **1** Yes, once or twice a week or more seldom,

0 Rarely or never

2. B: **3** Yes, several times a day, **2** Yes, several times a week, **1** Yes, once or twice a week or more seldom,

0 Rarely or never

2. C: **1** Several times a day, **0** Once a day, **2** Every second day, **3** Twice a week, **4** Once a week or more rarely

2. D: **0** Normal, **1** Diarrhoea-like, **2** Hard and dry

3.1 A1: **0** Contraception, **0** Reduction of menstruation pains, **0** Reduction of bleeding,

0 To regulate the menstrual cycle in relation to performances etc.., **1** Otherwise menstruation stops

3.2 A: **0** 11 years or younger, **0** 12-14 years, **1** 15 years or older, **0** I don't remember,

8 I have never menstruated

3.2 B: **0**Yes, **1** No, **1** I don't remember

3.2 B1: **1** Hormonal treatment, **1** Weight gain, **1** Reduced amount of exercise, **1** Other

3.2 C: **0** Yes, **2** No (go to question 3.2 C6), **1** I don't know (go to question 3.2 C6)

3.2 C1: **0** 0-4 weeks ago, **1** 1-2 months ago, **2** 3-4 months ago, **3** 5 months ago or more

3.2 C2: **0** Yes, most of the time, **1** No, mostly not

3.2 C3: **1** 1-2 days, **0** 3-4 days, **0** 5-6 days, **0** 7-8 days, **0** 9 days or more

3.2 C4: **0** Yes, **0** No

3.2 C5: **0** 12 or more, **1** 9-11, **2** 6-8, **3** 3-5, **4** 0-2

3.2 C6: **1** 2-3 months ago, **2** 4-5 months ago, **3** 6 months ago or more

0 I'm pregnant and therefore do not menstruate

3.2 D: **0** No, never, **1** Yes, it has happened before, **2** Yes, that's the situation now

3.2 E: **1** Yes, **0** No

3.2 E1: **1** I bleed less, **1** I bleed fewer days, **2** My menstruations stops, **0** I bleed more, **0** I bleed more days
