# Supplementary material for: The Low Energy Availability in Females Questionnaire (LEAF-Q) as a Useful Tool to Identify Female Triathletes at Risk for Menstrual Disorders Related to Low Energy Availability
Source: Nutrients. 2023 Jan 27;15(3):650. doi: 10.3390/nu15030650 (PMC9920150; doi:10.3390/nu15030650)
Supplement: Supplementary file 1 [file nutrients-15-00650-s001.zip › nutrients-2168140 - The LEAF-Questionnaire.pdf]

(Supplemental Digital Content 1)

The LEAF-Q

A questionnaire for female athletes

Department of Nutrition, Exercise and Sports
Life Science
University of Copenhagen
Denmark
Contact: Anna Melin, aot@life.ku.dk

The low energy availability in females questionnaire (LEAF –Q), focuses on physiological symptoms of insufficient energy intake. The following pages contain questions regarding injuries, gastrointestinal and reproductive function. We appreciate you taking the time to fill out the LEAF-Q and the reply will be treated as confidential.

Name: ____________________

Address: ____________________

____

E-mail: ____________________

Cell: __________

Profession: ____________________

Education: ____________________

Age: ______(years)

Height: ______(cm) Weight: ______(kg)

Your highest weight with your present height: ______ (kg)
(excluding pregnancy)

Your lowest weight with your present height: ______ (kg)

Do you smoke? Yes ☐ No ☐

Do you use any medication (excluding oral contraceptives)? Yes ☐ No ☐

If yes, what kind of medication?____________________

Your normal amount of training (average) – number of hours per week and what kind of exercise, such as running, swimming, bicycling, strength training, technique training etc.:

____

____

____

Comments or further information regarding exercise:____________________

____

____

1. Injuries

Mark the response that most accurately describes your situation

A: Have you had absences from your training, or participation in competitions during the last year due to injuries?

☐ No, not at all ☐ Yes, once or twice ☐ Yes, three or four times ☐ Yes, five times or more

A1: If yes, for how many days absence from training or participation in competition due to injuries have you had in the last year?

☐ 1-7 days ☐ 8-14 days ☐ 15-21 days ☐ 22 days or more

A2: If yes, what kind of injuries have you had in the last year? ______________________________

____

Comments or further information regarding injuries:______________________________

____

____

2. Gastro intestinal function

A: Do you feel gaseous or bloated in the abdomen, also when you do not have your period?

☐ Yes, several times a day ☐ Yes, several times a week

☐ Yes, once or twice a week or more seldom ☐ Rarely or never

B: Do you get cramps or stomach ache which cannot be related to your menstruation?

☐ Yes, several times a day ☐ Yes, several times a week

☐ Yes, once or twice a week or more seldom ☐ Rarely or never

C: How often do you have bowel movements on average?

☐ Several times a day ☐ Once a day ☐ Every second day

☐ Twice a week ☐ Once a week or more rarely

D: How would you describe your normal stool?

☐ Normal (soft) ☐ Diarrhoea-like (watery) ☐ Hard and dry

Comments regarding gastrointestinal function: ____________________

____

3. Menstrual function and use of contraceptives

3.1 Contraceptives

Mark the response that most accurately describes your situation

A: Do you use oral contraceptives?

☐ Yes ☐ No

A1: If yes, why do you use oral contraceptives?

☐ Contraception ☐ Reduction of menstruation pains ☐ Reduction of bleeding

☐ To regulate the menstrual cycle in relation to performances etc..

☐ Otherwise menstruation stops

☐ Other____

____

A2: If no, have you used oral contraceptives earlier?

☐ Yes ☐ No

A2:1 If yes, when and for how long?____

____

____

B: Do you use any other kind of hormonal contraceptives? (e.g. hormonal implant or coil)

☐ Yes ☐ No

B1: If yes, what kind?

☐ Hormonal patches ☐ Hormonal ring ☐ Hormonal coil ☐ Hormonal implant ☐ Other

____

3.2 Menstrual function

Mark the response that most accurately describes your situation

A: How old were when you had your first period?

☐ 11 years or younger ☐ 12-14 years ☐ 15 years or older ☐ I don't remember

☐ I have never menstruated (If you have answered "I have never menstruated" there are no further questions to answer)

B: Did your first menstruation come naturally (by itself)?

☐ Yes ☐ No ☐ I don't remember

B1: If no, what kind of treatment was used to start your menstrual cycle?

☐ Hormonal treatment ☐ Weight gain

☐ Reduced amount of exercise ☐ Other

C: Do you have normal menstruation?

☐ Yes ☐ No **(go to question C6)** ☐ I don't know **(go to question C6)**

C1: If yes, when was your last period?

☐ 0-4 weeks ago ☐ 1-2 months ago ☐ 3-4 months ago ☐ 5 months ago or more

C2: If yes, are your periods regular? (Every 28th to 34th day)

☐ Yes, most of the time ☐ No, mostly not

C3: If yes, for how many days do you normally bleed?

☐ 1-2 days ☐ 3-4 days ☐ 5-6 days ☐ 7-8 days ☐ 9 days or more

C4: If yes, have you ever had problems with heavy menstrual bleeding?

☐ Yes ☐ No

C5: If yes, how many periods have you had during the last year?

☐ 12 or more ☐ 9-11 ☐ 6-8 ☐ 3-5 ☐ 0-2

3.2 Menstrual function

Mark the response that most accurately describes your situation

C6: If no or "I don't remember", when did you have your last period?

- ☐ 2-3 months ago
- ☐ 4-5 months ago
- ☐ 6 months ago or more
- ☐ I'm pregnant and therefore do not menstruate

D: Have your periods ever stopped for 3 consecutive months or longer (besides pregnancy)?

- ☐ No, never
- ☐ Yes, it has happened before
- ☐ Yes, that's the situation now

E: Do you experience that your menstruation changes when you increase your exercise intensity, frequency or duration?

- ☐ Yes
- ☐ No

E1: If yes, how? (Check one or more options)

- ☐ I bleed less
- ☐ I bleed fewer days
- ☐ My menstruations stops
- ☐ I bleed more
- ☐ I bleed more days
